# Detection of *Helicobacter pylori* Microevolution and Multiple Infection from Gastric Biopsies by Housekeeping Gene Amplicon Sequencing

**DOI:** 10.3390/pathogens9020097

**Published:** 2020-02-05

**Authors:** Montserrat Palau, Núria Piqué, André M. Comeau, Gavin M. Douglas, M. José Ramírez-Lázaro, Sergio Lario, Xavier Calvet, Morgan G. I. Langille, David Miñana-Galbis

**Affiliations:** 1 Secció de Microbiologia, Departament de Biologia, Sanitat i Medi Ambient, Facultat de Farmàcia i Ciències de l’Alimentació, Universitat de Barcelona, Av. Joan XXIII 27-31, 08028 Barcelona, Catalonia, Spain; montsepalau.8@gmail.com (M.P.); npique@ub.edu (N.P.); 2 Integrated Microbiome Resource (IMR), Dalhousie University, 5850 College Street, Halifax, NS B3H 4R2, Canada; Andre.Comeau@dal.ca (A.M.C.); morgan.g.i.langille@dal.ca (M.G.I.L.); 3 Department of Microbiology & Immunology, Dalhousie University, Halifax, NS B3H 4R2, Canada; gavin.douglas@dal.ca; 4 Digestive Diseases Service, Hospital de Sabadell, Institut Universitari Parc Taulí-UAB, Parc Tauli 1, 08208 Sabadell, Catalonia, Spain; MRamirezL@tauli.cat (M.J.R.-L.); slario@outlook.es (S.L.); xcalvet@tauli.cat (X.C.); 5 Centro de Investigación Biomédica en Red de Enfermedades Hepáticas y Digestivas (CIBERehd), Instituto de Salud Carlos III, Montorte de Lemos 3–5, 28029 Madrid, Spain; 6 Department of Pharmacology, Dalhousie University, 5850 College Street, Halifax, NS B3H 4R2, Canada

**Keywords:** *Helicobacter pylori*, microevolution, multiple infections, housekeeping genes, amplicon sequencing, gastric diseases

## Abstract

Despite the great efforts devoted to research on *Helicobacter pylori*, the prevalence of single-strain infection or *H. pylori* mixed infection and its implications in the mode of transmission of this bacterium are still controversial. In this study, we explored the usefulness of housekeeping gene amplicon sequencing in the detection of *H. pylori* microevolution and multiple infections. DNA was extracted from five gastric biopsies from four patients infected with distinct histopathological diagnoses. PCR amplification of six *H. pylori*-specific housekeeping genes was then assessed on each sample. Optimal results were obtained for the *cgt* and *luxS* genes, which were selected for amplicon sequencing. A total of 11,833 *cgt* and 403 *luxS* amplicon sequences were obtained, 2042 and 112 of which were unique sequences, respectively. All *cgt* and *luxS* sequences were clustered at 97% to 9 and 13 operational taxonomic units (OTUs), respectively. For each sample from a different patient, a single OTU comprised the majority of sequences in both genes, but more than one OTU was detected in all samples. These results suggest that multiple infections with a predominant strain together with other minority strains are the main way by which *H. pylori* colonizes the human stomach.

## 1. Introduction

*Helicobacter pylori* is a Gram-negative bacterium characterized by a high level of intraspecies genetic diversity. This species has been intensely investigated with more than 43,000 research articles cited in PubMed (https://www.ncbi.nlm.nih.gov/pubmed/). One reason for such interest is that *H. pylori* has evolved with humans for about 100,000 years. Having coexisted with humans for this long period of time, tracing human migrations is still possible today through multilocus sequence analysis (MLSA) of housekeeping genes of *H. pylori* strains isolated from human populations with distinct geographical origins [1,2].

Humans and some other primates are the unique hosts of *H. pylori*, which infects gastric mucosa as the primary niche and persists for the lifetime of the host in the absence of treatment. *H. pylori* infects about half of the world’s population and the infection is mainly acquired in early childhood [3,4]. Infection results in life-long gastric colonization that usually is asymptomatic. However, it can lead to the development of several gastro-intestinal diseases, such as gastric ulcers, mucosa-associated lymphoid tissue (MALT) lymphoma, or chronic inflammation. The latter could potentially progress to multifocal atrophy, intestinal metaplasia, dysplasia, and gastric adenocarcinoma [5,6,7,8].

It has been suggested that the transmission of *H. pylori* occurs directly via oral–oral, gastro–oral, and fecal–oral routes, although food- and water-borne transmissions could play an important role [7,9]. Another controversial issue related to transmission is the prevalence of *H. pylori* infection whether by a single strain or by multiple strains, since different methodologies and discordant results have been reported [10,11]. Studies of *H. pylori* infection at strain level using high-throughput analyses could be key to elucidate the emergence or transmission of antibiotic resistance and the development of gastric diseases.

In addition to the housekeeping genes (*atpA*, *efp*, *mutY*, *ppa*, *trpC*, *ureI*, and *yphC*) used in MLSA studies to delineate *H. pylori*/human populations previously described [1], we have recently developed *H. pylori* specific PCR amplifications of six housekeeping genes (*amiA*, *cgt*, *cpn60*, *cpn70*, *dnaJ*, and *luxS*) related to *H. pylori* pathogenesis in order to evaluate their usefulness as new virulence markers and for the specific detection of *H. pylori*, the genetic discrimination at strain level and the detection of multiple infection [11].

The aim of this study was to evaluate the usefulness of amplicon sequencing methodology for the detection of *H. pylori* microevolution and multiple infections from gastric biopsies of patients with dyspeptic symptoms and different histopathological findings (from atrophy to adenocarcinoma).

## 2. Results

### 2.1. Samples and Amplicon Sequences

As the best PCR amplification of six housekeeping genes (*amiA*, *cgt*, *cpn60*, *cpn70*, *dnaJ*, and *luxS*) from DNA biopsies were obtained for the genes *cgt* and *luxS*, these genes were selected for amplicon sequencing (see Materials and Methods). The sequencing results of the amplicon libraries (Table 1 and Table 2) yielded a much larger number of *cgt* sequences (11,833 in total and 2042 unique sequences) than *luxS* sequences (403 in total and 112 unique sequences). This is probably due to the increased dilution of *luxS* amplicons in the preparation of the pooling. All sequences from each sample and gene were processed in order to adjust for the length of the sequences (438 nt for *cgt* sequences and 366 nt for *luxS* sequences) and to detect duplicate sequences. As a result, a significant number of identical sequences and a low number of unique sequences (11–43%) were obtained (Table 1 and Table 2).

The distribution of total and unique *cgt* sequences by sample was as follows (Table 1): 5185 total and 593 (11%) unique sequences from sample B247S; 2422 and 422 (17%) from B373A; 968 and 234 (24%) from B508S; 1414 and 339 (24%) from B508T; and 1844 and 454 (25%) from B601A. In the case of *luxS*, the distribution of total and unique sequences by sample was as follows (Table 2): 111 total and 34 (31%) unique sequences from sample B247S; 72 and 16 (22%) from B373A; 56 and 18 (32%) from B508S; 115 and 23 (20%) from B508T; and 49 and 21 (43%) from B601A.

### 2.2. Operational Taxonomic Units (OTUs) Assignment and Distribution

All unique sequences belonging to the same gene were merged in a FASTA file and pairwise distances were calculated to generate a PHYLIP-formatted distance matrix. A cutoff distance of 3% for both genes was defined in order to assign sequences to operational taxonomic units (OTUs). In a previous study [11], it was shown that for the *luxS* gene this value was the mean distance value between sequences from clones isolated from different patients. The distant value of the *cgt* gene was calculated to be 1%. However, due to the inherent error rates of Roche 454 platform, it was decided to raise the value to 3%. Therefore, sequences with distance <3% were considered as originating from the same strain. A total of 9 and 13 OTUs were obtained from the *cgt* (Table 1) and *luxS* (Table 2) distance matrix, respectively. In all samples, at least two different OTUs were detected. Additionally, most of the total (99% from *cgt* and 91% from *luxS*) and unique (98% from *cgt* and 73% from *luxS*) sequences clustered in OTUs 01–04. Some sequences from different samples belonged to the same OTU, especially in the case of OTU-03 (containing sequences from samples B508S and B508T) (Table 1 and Table 2).

As seen in the tables, every sample had a dominant OTU. For example, for the gene *cgt* in the sample B247S, 5184 sequences out of 5185 of the total were in OTU-01. When referring to unique sequences, 592 of 593 are in this OTU. Regarding the gene *luxS* in the same sample, 98 sequences of a total of 111 were in OTU-01. And referring to unique sequences, 25 of a total of 34 were in this OTU.

### 2.3. Amplicon Sequence Identification

As *cgt* and *luxS* PCRs were specific for *H. pylori*, all amplicon sequences matched this species. In order to classify the amplicon sequences obtained in this study, two FASTA-formatted sequence files for each gene were generated. One file contained the representative sequence for each OTU (by means of the *get.oturep* Mothur command) and the other file with reference sequences as detailed below.

For the reference sequences file, *cgt* and *luxS* sequences were downloaded from 75 *H. pylori* complete genomes hosted in GenBank (Supplementary Table S1). These sequences were processed using Mothur (*unique.seqs* command) in order to avoid duplicate sequences, resulting in 53 *cgt* and 59 *luxS* unique sequences. An additional 15 *cgt* and *luxS* sequences were also incorporated as reference sequences (Supplementary Table S1). These additional sequences had been previously described in Palau et al. [11]. Furthermore, some of them were obtained from strains isolated from the same biopsies (B247S, B373A, B508S, and B508T) used in this study. All reference sequences were adjusted to the length of 438 nt for *cgt* and 366 nt for *luxS*.

Representative OTU sequences from this study were classified using Mothur (*classify.seqs* command) with the above reference sequences as templates. On the other hand, all sequences (representative OTUs and references) were joined in a single file, one for each gene. Thereafter, the sequence distances were calculated and phylogenetic trees were constructed using MEGA7 [12]. Moreover, representative OTU sequences were also compared using Nucleotide BLAST on the NCBI website.

**Figure 1:**
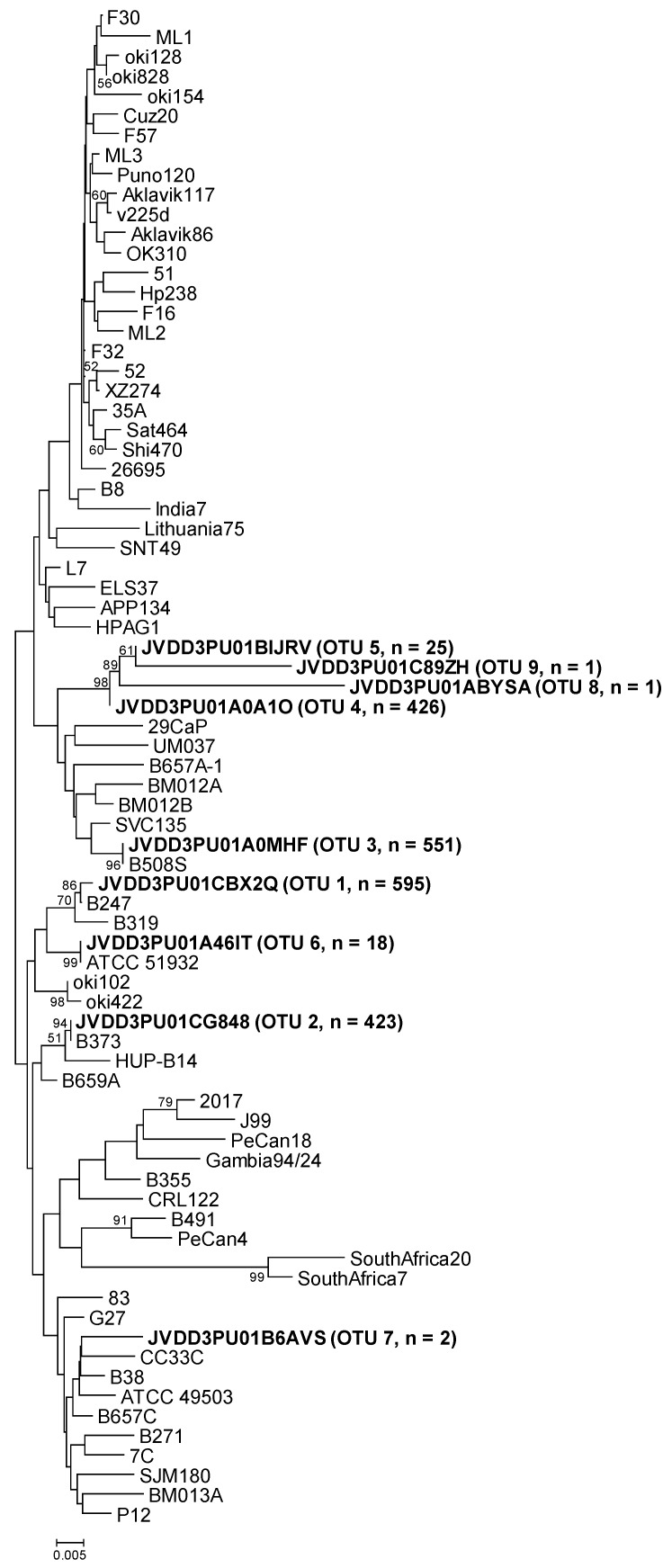
Neighbor-joining phylogenetic tree obtained from representative OTU sequences and reference sequences of gene *cgt*. Bar, distance values as calculated by MEGA 7.0. Bootstrap values (> 50%) after 1000 replicates are shown as percentages.

The representative sequence of *cgt* OTU-01, obtained from sample B247S, showed one nucleotide difference (the first nucleotide of the sequence) with respect to the *cgt* reference sequence (GenBank accession number KU053362). The reference was obtained from *H. pylori* strain B247 (Table 3 and Figure 1), which was isolated from replicate biopsy B247S in a previous study [11]. The representative sequence of *cgt* OTU-02 (from sample B373A) was identical to the *cgt* reference sequence (GenBank accession number MG950173). In this case, the reference was obtained from *H. pylori* strain B373, isolated from replicate biopsy B373A in the above-mentioned study. The representative sequence of *cgt* OTU-03, that comprised most of the sequences from samples B508S and B508T, was identical to the *cgt* reference sequence that was obtained from *H. pylori* strain B508S (GenBank accession number KU053367), isolated from replicate biopsy B508S. The representative sequence of the other principal OTU (OTU-04) (from sample B601A) could not be compared to the corresponding strain sequence because no *H. pylori* strain was isolated from biopsy B601A. The other five *cgt* OTUs were different from any reference sequence, except in the case of *cgt* OTU-06. Its sequence was identical to the sequence of *H. pylori* ATCC 51932 (Table 3 and Figure 1).

**Figure 2:**
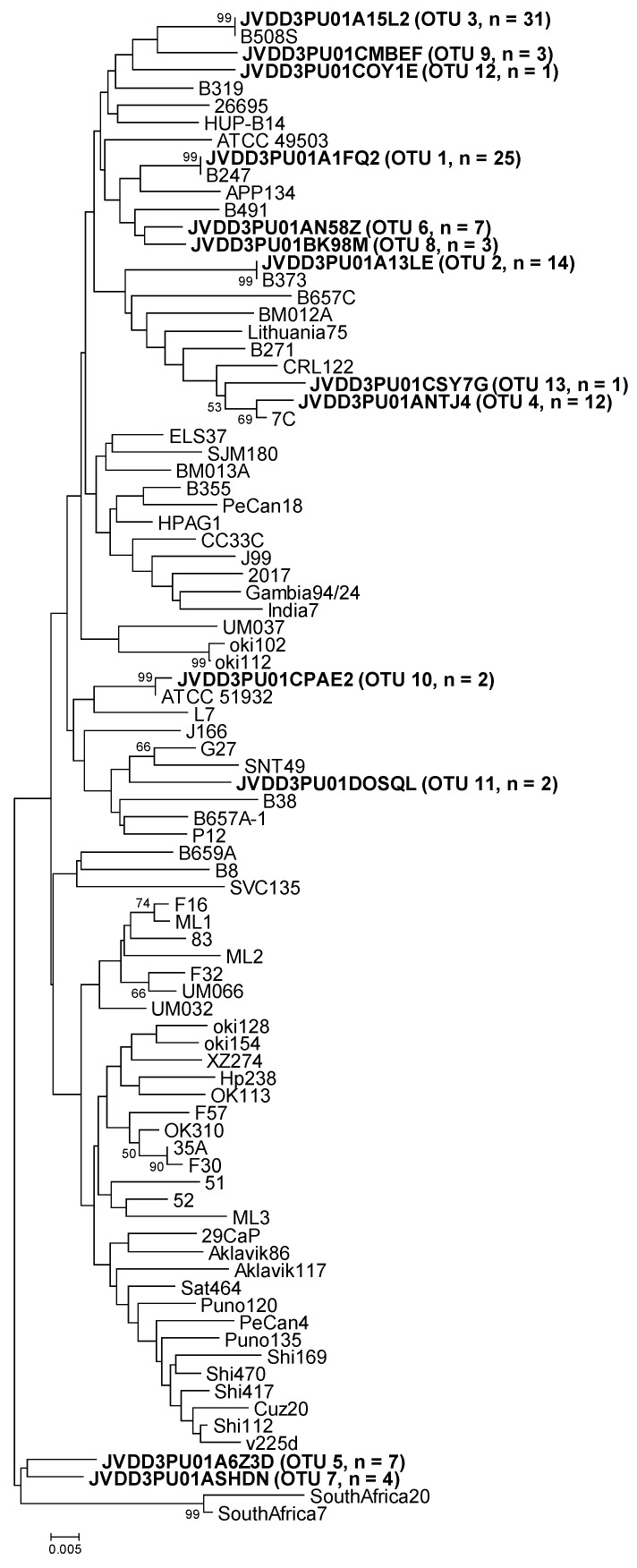
Neighbor-joining phylogenetic tree obtained from representative OTU sequences and reference sequences of gene *luxS*. Bar, distance values as calculated by MEGA 7.0. Bootstrap values (>50%) after 1000 replicates are shown as percentages.

Similarly, the representative sequences of *luxS* OTU-01, -02, and -03 were identical to the reference sequences (GenBank accession numbers KU053434, MG950172, and KU053439, respectively) that were obtained from the above-mentioned strains, isolated from replicate gastric biopsies of the same patient (B247, B373, and B508, respectively) (Table 4 and Figure 2). The representative sequence of *luxS* OTU-04 could not be compared either to the corresponding strain sequence because of the lack of *H. pylori* strain isolation from biopsy B601A. The other nine *luxS* OTUs were different from any reference sequence (Table 4 and Figure 2).

## 3. Discussion

Most amplicon sequences were repeated sequences that were largely represented by four OTUs. Representative sequences from these OTUs matched with sequences of strains isolated from replicate gastric biopsies obtained from the same patient (except in the case of sample B601A because of the lack of the corresponding isolate). However, although in the minority, other OTUs were detected in all five gastric biopsies for both genes. These results suggest that *H. pylori* colonizes the human stomach through different infection events that lead to a gastric multi-infection with a predominant strain together with other minority strains.

Other studies have evaluated the prevalence of mixed *H. pylori* infections, but with a high disparity of results, even when using similar methodologies. Using randomly amplified polymorphic DNA (RAPD) fingerprinting, Toita et al. [13] reported that all patients were infected with a single *H. pylori* clone. Contrarily, Sheu et al. [14] found a 23.3% prevalence of mixed infections. Analyzing the *cag*-PAI status and the s-region or m-region of *vacA* in *H. pylori* isolates by PCR, Lai et al. [15] described a prevalence of 28% for mixed infections. Additionally, Kibria et al. [16] studied the prevalence of mixed *H. pylori* infection using both methods mentioned above (RAPD fingerprinting and multiplex PCR amplification for *cagA* and *vacA* alleles). An overall prevalence of 60.2% was obtained. Recently, an event of multiple infection was detected from 52 *H. pylori* clones isolated from 11 patients by sequencing of six housekeeping genes (*amiA*, *cgt*, *cpn60*, *cpn70*, *dnaJ*, and *luxS*) [11]. However, Raymond et al. [17] detected mixed infection in all six members of a family analyzing 107 clones by multilocus sequence typing (MLST) of two housekeeping genes (*hspA* and *glmM*).

To our knowledge, the present work is the first study that uses amplicon sequencing of housekeeping genes to detect *H. pylori* multi-infections. Amplicon sequencing is a high-throughput technique with greater resolution when compared to other methods (i.e., RAPD fingerprinting, multiplex *cagA* and *vacA* PCR, and MLST) used in previous studies. In the present study, multiple *H. pylori* infections with a predominant strain were detected in all the gastric biopsies analyzed in accordance with results obtained for Raymond et al. [17]. Although further studies are needed to confirm our results, these suggest that mixed *H. pylori* infections are the main status in the colonization of the human gastric mucosa. In fact, a high frequency of recombination between unrelated strains in mixed colonization is the main cause of the panmictic population structure of *H. pylori* [18,19,20]. In this study, microevolution by mutation of original strains infecting the gastric mucosa [19,20,21] was also found, since different unique sequences were obtained in each OTU (if consisted of two or more amplicon sequences).

Amplicon sequencing of housekeeping, virulence or antibiotic-resistant genes could be useful in different fields. It could be useful for elucidating the mode of transmission of *H. pylori*, to perform epidemiological studies. The impact of mixed *H. pylori* infection in the gastric pathogenesis and the failure of antibiotic treatments also deserves additional investigation.

In conclusion, our study strongly suggests that multiple infections including a predominant strain and multiple minority *H. pylori* is the predominant pattern of *H. pylori* infections in humans. The clinical and biological relevance of this finding deserves further study.

## 4. Materials and Methods 

### 4.1. Gastric Biopsies

Patient recruitment and gastric biopsies obtained by the Digestive Diseases Department of the Hospital Taulí (Sabadell, Catalonia, Spain) were described in previous studies [11,22]. The study was approved by the Ethics Committee in accordance with the Declaration of Helsinki. Biopsies were performed after obtaining the patients’ written informed consent. For the present study, DNA from five gastric biopsies corresponding to four different patients infected with *H. pylori* were analyzed. Regarding the patients, two suffered from gastric cancer (B247, B508), one from duodenal ulcer (B373), and one was without medical conditions, here labeled as normal stomach (B601). Histological diagnoses from the biopsies were: chronic active gastritis, atrophy, intestinal metaplasia, and adenocarcinoma. Biopsies B508S and B508T were taken from the same patient with gastric adenocarcinoma (B508). Specifically, biopsy B508S was taken from normal gastric tissue and biopsy B508T from the tumoral tissue (Table 5).

### 4.2. DNA Extraction and PCR Amplification

Extraction and sequential purification of DNA were performed using the MasterPure kit (Epicentre, Illumina). Isolated DNA was quantified with a NanoDrop spectrophotometer (Nano-Drop Technologies, USA).

Using *H. pylori* specific primers, PCR amplifications of six housekeeping genes (*amiA*, *cgt*, *cpn60*, *cpn70*, *dnaJ*, and *luxS*) were performed as described in a previous work of the authors [11], in which these genes were useful for *H. pylori* clone’s discrimination. From these genes, *cgt* and *luxS* demonstrated the best PCR results (a single, clear band with the corresponding length revealed in agarose gel) and therefore, were selected for amplicon sequencing.

Fusion *cgt* and *luxS* primers (Table 6) were designed for unidirectional sequencing following the 454 sequencing system guidelines for amplicon experimental design (454 Life Sciences Corp., Branford, CT, USA). PCR amplifications were carried out with the FastStart High Fidelity PCR System (Roche Diagnostics GmBH, Mannheim, Germany) in a total volume of 50 µL containing 2.5 mM MgCl_2_, 0.2 mM dNTPs, 2.5 U FastStart polymerase, 40 pmol of each primer (Isogen Life Science, PW De Meern, The Netherlands), and 200 ng DNA. The reaction mixtures were subjected to the following thermal cycling program in a PrimeG Thermal Cycler (Bibby Scientific, Staffordshire, UK): initial denaturation at 95 °C for 5 min; 35 cycles of 95 °C for 1 min, 60 °C (*cgt*) or 54 °C (*luxS*) for 1 min, and 72 °C for 1 min; final extension at 72 °C for 5 min. The amplified products were purified using ExoSAP-IT^®^ kit (Affymetrix, Santa Clara, CA, USA).

### 4.3. Preparation of Libraries

Following the Amplicon Library Preparation Manual (454 Life Sciences Corp., Branford, CT, USA), the amplicons including fusion primers of each sample were sequenced using the 454 GS Junior instrument (Roche, Basel, Switzerland). After PCR purifications, library quantification was done using a Qubit^TM^ Fluorometer (Thermo Fisher Scientific, Waltham, MA, USA). Each amplicon was diluted separately to 1 × 10^9^ molecules/µL in a 1xTE buffer. Diluted amplicons were mixed to prepare the amplicon pool. As the size of *cgt* and *luxS* amplicons were different (685 bp for *cgt* and 499 bp for *luxS*), mixed volumes were adjusted to the ratio 1.2:1 *cgt*:*luxS*. The amplicon pool was diluted to 1 × 10^7^ molecules/µL in molecular biology grade water. Once the libraries were constructed and pooled, emPCR amplification and sequencing were performed in the facilities of the Genomics Unit of Scientific and Technological Centers from the University of Barcelona (CCiTUB).

### 4.4. Data Processing

Raw data were processed by the GS Run Processor, which resulted in FASTQ and Standard Flowgram Format (SFF) files that were used in subsequent analysis. Amplicon sequences were assigned to each corresponding sample using the multiplex identifiers (MIDs), generating five files for each gene (Table 6). Thereafter, MIDs, sequences <50 bp or containing >5% Ns were removed from FASTQ files [23]. This processing was carried out by the CCiTUB.

Sequences in FASTA files were aligned with *cgt* or *luxS* reference sequences [11] using the MEGA7 software [12]. The aim was to check that amplicon sequences corresponded to *cgt* or *luxS* sequences. As mentioned above (see Section 2.3), some reference sequences corresponded to *H. pylori* strains isolated previously from the gastric biopsies B247, B373, and B508; replicate biopsies of the same patient at the same time were used in the present study. Additionally, in this process, the length of the sequences was adjusted. All sequences were deposited in Genbank under the project accession number PRJNA434670.

Sequences were analyzed with Mothur [24] following the subsequent protocol (all commands were run with the default parameters except when explicitly specified): first, the *unique.seqs* command was used in order to obtain the unique sequences of the sequence file by dereplication. Next, the *merge.files* command concatenates multiple files, which is useful for merging multiple FASTA-formatted sequence files. The *make.group* command reads a series of FASTA files and creates a group file, which is used to assign sequences to a specific group. The *dist.seqs* command then calculates uncorrected pairwise distances between aligned DNA sequences. By default, *dist.seqs* penalizes terminal gaps. This option was excluded and the default value of *countends* was changed to False. Afterwards, the *cluster* command was used to assign sequences to OTUs; the *furthest neighbor* clustering method was chosen, as it is the most conservative way to select OTUs. The *make.shared* and the *summary.shared* commands were used successively to obtain a summary file with the calculated values for each line in the OTU data and for all possible comparisons between the different groups in the group file. The *get.oturep* command generated a FASTA-formatted sequence file containing only one representative sequence for each OTU. Finally, the *classify.seqs* command was used to taxonomically classify the sequences.

## Figures and Tables

**Table 1:** Distribution of *cgt* amplicon sequences by samples and operational taxonomic units (OTUs).

*cgt* OTUs	B247S Sequences		B373A Sequences		B508S Sequences		B508T Sequences		B601A Sequences		*cgt* Sequences	
	Total	Unique	Total	Unique	Total	Unique	Total	Unique	Total	Unique	Total	Unique
OTU-01	5184	592	−	−	−	−	3	3	−	−	5187	595
OTU-02	1	1	2416	418	−	−	4	4	−	−	2421	423
OTU-03	−	−	−	−	963	230	1391	321	−	−	2354	551
OTU-04	−	−	−	−	−	−	−	−	1808	426	1808	426
OTU-05	−	−	−	−	−	−	−	−	30	25	30	25
OTU-06	−	−	6	4	4	3	16	11	−	−	26	18
OTU-07	−	−	−	−	1	1	−	−	4	1	5	2
OTU-08	−	−	−	−	−	−	−	−	1	1	1	1
OTU-09	−	−	−	−	−	−	−	−	1	1	1	1
total	5185	593 (11%)	2422	422 (17%)	968	234 (24%)	1414	339 (24%)	1844	454 (25%)	11,833	2042 (17%)

**Table 2:** Distribution of *luxS* amplicon sequences by samples and OTUs.

*luxS* OTUs	B247S Sequences		B373A Sequences		B508S Sequences		B508T Sequences		B601A Sequences		*luxS* Sequences	
	Total	Unique	Total	Unique	Total	Unique	Total	Unique	Total	Unique	Total	Unique
OTU-01	98	25	−	−	−	−	−	−	−	−	98	25
OTU-02	−	−	70	14	−	−	−	−	−	−	70	14
OTU-03	−	−	−	−	52	14	107	15	5	2	164	31
OTU-04	−	−	−	−	−	−	−	−	33	12	33	12
OTU-05	−	−	−	−	2	2	1	1	8	4	11	7
OTU-06	7	6	−	−	−	−	1	1	−	−	8	7
OTU-07	−	−	2	2	−	−	1	1	1	1	4	4
OTU-08	6	3	−	−	−	−	−	−	−	−	6	3
OTU-09	−	−	−	−	1	1	2	2	−	−	3	3
OTU-10	−	−	−	−	−	−	2	2	−	−	2	2
OTU-11	−	−	−	−	1	1	1	1	−	−	2	2
OTU-12	−	−	−	−	−	−	−	−	1	1	1	1
OTU-13	−	−	−	−	−	−	−	−	1	1	1	1
total	111	34 (31%)	72	16 (22%)	56	18 (32%)	115	23 (20%)	49	21 (43%)	403	112 (28%)

**Table 3:** Classification of *cgt* OTUs by Mothur, MEGA7, and BLAST.

*cgt* OTUs	Representative Sequence	Mothur	MEGA7 (% Similarity)	BLAST (% Similarity)
OTU-01	JVDD3PU01CBX2Q	B247	B247 (99.8)	B247 (100)
OTU-02	JVDD3PU01CG848	B373A	B373A (100)	B373A (100)
OTU-03	JVDD3PU01A0MHF	B508S	B508S (100)	B508S (100)
OTU-04	JVDD3PU01A0A1O	SVC135	OTU-05 (99.5), SVC135 (98.6)	SVC135 (99)
OTU-05	JVDD3PU01BIJRV	SVC135	OTU-04 (99.5), SVC135 (98.2)	SVC135 (98)
OTU-06	JVDD3PU01A46IT	ATCC 51932	ATCC 51932 (100)	ATCC 51932 (100)
OTU-07	JVDD3PU01B6AVS	B508S	B508S, B657A-4, B38 and OTU-03 (98.4)	B508S, B657A-4 and B38 (98)
OTU-08	JVDD3PU01ABYSA	SVC135	OTU-04 (95.8), SVC135 (94.3)	SVC135 (98)
OTU-09	JVDD3PU01C89ZH	SVC135	OTU-05 (97.2), OTU-04 (96.7), SVC135 (95.3)	SVC135 (97)

**Table 4:** Classification of *luxS* OTUs by Mothur, MEGA7, and BLAST.

*luxS* OTUs	Representative Sequence	Mothur	MEGA7 (% Similarity)	BLAST (% Similarity)
OTU-01	JVDD3PU01A1FQ2	B247	B247 (100)	B247 (100)
OTU-02	JVDD3PU01A13LE	B373A	B373A (100)	B373A (100)
OTU-03	JVDD3PU01A15L2	B508S	B508S (100)	B508S (100)
OTU-04	JVDD3PU01ANTJ4	7C	7C (99.2)	7C (99)
OTU-05	JVDD3PU01A6Z3D	oki112	OTU-07 (97.8), OTU-06, B247 and B319 (96.9)	B247 and B319 (97)
OTU-06	JVDD3PU01AN58Z	B247	OTU-08 (98.6), B247 and OTU-02 (98.3)	B247 (98)
OTU-07	JVDD3PU01ASHDN	B373A	OTU-05 (97.8), ELS37 (97.2)	ELS37 (97)
OTU-08	JVDD3PU01BK98M	B491	OTU-06 (98.6), B319 and B491 (97.5)	B319, B491 and ATCC 51932 (97)
OTU-09	JVDD3PU01CMBEF	B508S	B508S and OTU-03 (97.2)	B508S (98)
OTU-10	JVDD3PU01CPAE2	ATCC 51932	ATCC 51932 (99.7)	ATCC 51932 (99)
OTU-11	JVDD3PU01DOSQL	G27	G27 (97.2)	G27 (97)
OTU-12	JVDD3PU01COY1E	7C	OTU-04 (97.5), 7C, B319, B508S, ELS37, HPAG1, HUP-B14 and OTU-03 (96.6)	7C, B319, B508S, ELS37, HPAG1 and HUP-B14 (97)
OTU-13	JVDD3PU01CSY7G	B508S	OTU-04 (97.8), 7C (97.5)	7C (98)

**Table 5:** Samples included in this study.

Patient ID	Age and Gender	Endoscopic Diagnosis	Anatomical Location of Biopsy Specimen	Histopathological Diagnosis *	Specimen ID
B247	63 y/o male	Gastric cancer	Non-neoplastic stomach tissue	Chronic active gastritis	B247S
B373	56 y/o male	Duodenal ulcer	Antrum	Intestinal metaplasia	B373A
B508	77 y/o male	Gastric cancer	Non-neoplastic stomach tissue	Not available	B508S
			Tumor tissue	Adenocarcinoma	B508T
B601	43 y/o female	Normal	Antrum	Atrophy	B601A

* Most advanced lesion observed.

**Table 6:** Fusion primers designed for *H. pylori* specific PCR and amplicon sequencing.

Gene	Fusion Primer	Sequence (Adaptor–*key*–MID–Template-Specific Sequence)
*cgt*	cgt-252-B247S	CCATCTCATCCCTGCGTGTCTCCGAC*TCAG***AGACGCACTC**GGCTTTTAAGGGAGCGGATA
	cgt-252-B373A	CCATCTCATCCCTGCGTGTCTCCGAC*TCAG***AGCACTGTAG**GGCTTTTAAGGGAGCGGATA
	cgt-252-B508S	CCATCTCATCCCTGCGTGTCTCCGAC*TCAG***ACGAGTGCGT**GGCTTTTAAGGGAGCGGATA
	cgt-252-B508T	CCATCTCATCCCTGCGTGTCTCCGAC*TCAG***ACGCTCGACA**GGCTTTTAAGGGAGCGGATA
	cgt-252-B601A	CCATCTCATCCCTGCGTGTCTCCGAC*TCAG***ATCAGACACG**GGCTTTTAAGGGAGCGGATA
	cgt-866 (reverse)	CCTATCCCCTGTGTGCCTTGGCAGTC*TCAG*ATCGCTTCGCTYTCCACATT
*luxS*	luxS-38-B247S	CCATCTCATCCCTGCGTGTCTCCGAC*TCAG***AGACGCACTC**TGGATCACACYAAAGTCAAAG
	luxS-38-B373A	CCATCTCATCCCTGCGTGTCTCCGAC*TCAG***AGCACTGTAG**TGGATCACACYAAAGTCAAAG
	luxS-38-B508S	CCATCTCATCCCTGCGTGTCTCCGAC*TCAG***ACGAGTGCGT**TGGATCACACYAAAGTCAAAG
	luxS-38-B508T	CCATCTCATCCCTGCGTGTCTCCGAC*TCAG***ACGCTCGACA**TGGATCACACYAAAGTCAAAG
	luxS-38-B601A	CCATCTCATCCCTGCGTGTCTCCGAC*TCAG***ATCAGACACG**TGGATCACACYAAAGTCAAAG
	luxS-466 (reverse)	CCTATCCCCTGTGTGCCTTGGCAGTC*TCAG*AAACCCCCACTTCAGACCA

## Supplementary Materials

The following are available online at https://www.mdpi.com/2076-0817/9/2/97/s1, Table S1: Reference sequences used in this study.

## References

[1] Moodley Y., Linz B., Bond R.P., Nieuwoudt M., Soodyall H., Schlebusch C.M., Bernhöft S., Hale J., Suerbaum S., Mugisha L. (2012). Age of the association between *Helicobacter pylori* and man. PLoS Pathog..

[2] Suzuki R., Shiota S., Yamaoka Y. (2012). Molecular epidemiology, population genetics, and pathogenic role of *Helicobacter pylori*. Infect. Genet. Evol..

[3] Piqué N., Palau M., Berlanga M., Miñana-Galbis D. (2016). Advances in the research of new genetic markers for the detection of *Helicobacter pylori* infection. Recent Advances in Pharmaceutical Sciences VI.

[4] Hashi K., Imai C., Yahara K., Tahmina K., Hayashi T., Azuma T., Miyabe-Nishiwaki T., Sato H., Matsuoka M., Niimi S. (2018). Evaluating the origin and virulence of a *Helicobacter pylori cagA*-positive strain isolated from a non-human primate. Sci. Rep..

[5] Correa P., Houghton J. (2007). Carcinogenesis of *Helicobacter pylori*. Gastroenterology.

[6] Buzás G.M. (2018). Benign and malignant gastroduodenal diseases associated with *Helicobacter pylori*: A narrative review and personal remarks in 2018. Minerva Gastroenterol. Dietol..

[7] Quaglia N.C., Dambrosio A. (2018). *Helicobacter pylori*: A foodborne pathogen?. World J. Gastroenterol..

[8] Kabamba E.T., Tuan V.P., Yamaoka Y. (2018). Genetic populations and virulence factors of *Helicobacter pylori*. Infect. Genet. Evol..

[9] Smith S., Fowora M., Pellicano R. (2019). Infections with *Helicobacter pylori* and challenges encountered in Africa. World J. Gastroenterol..

[10] Ben Mansour K., Fendri C., Battikh H., Garnier M., Zribi M., Jlizi A., Burucoa C. (2016). Multiple and mixed *Helicobacter pylori* infections: Comparison of two epidemiological situations in Tunisia and France. Infect. Genet. Evol..

[11] Palau M., Kulmann M., Ramírez-Lázaro M.J., Lario S., Quílez M.E., Campo R., Piqué N., Calvet X., Miñana-Galbis D. (2016). Usefulness of housekeeping genes for the diagnosis of *Helicobacter pylori* infection, strain discrimination and detection of multiple infection. Helicobacter.

[12] Kumar S., Stecher G., Tamura K. (2016). MEGA7: Molecular Evolutionary Genetics Analysis Version 7.0 for Bigger Datasets. Mol. Biol. Evol..

[13] Toita N., Yokota S., Fujii N., Konno M. (2013). Clonality Analysis of *Helicobacter pylori* in patients isolated from several biopsy specimens and gastric juice in a Japanese urban population by random amplified polymorphic DNA fingerprinting. Gastroenterol. Res. Pract..

[14] Sheu S.M., Sheu B.S., Lu C.C., Yang H.B., Wu J.J. (2009). Mixed infections of *Helicobacter pylori*: Tissue tropism and histological significance. Clin. Microbiol. Infect..

[15] Lai C.H., Huang J.C., Chiang-Ni C., Li J.-P., Wu L.-T., Wu H.-S., Sun Y.-C., Lin M.-L., Lee J.-F., Lin H.-J. (2016). Mixed infections of *Helicobacter pylori* isolated from patients with gastrointestinal diseases in Taiwan. Gastroenterol. Res. Pract..

[16] Kibria K.M., Hossain M.E., Sultana J., Sarker S.A., Bardhan P.K., Rahman M., Nahar S. (2015). The prevalence of mixed *Helicobacter pylori* infections in symptomatic and asymptomatic subjects in Dhaka, Bangladesh. Helicobacter.

[17] Raymond J., Thiberg J.M., Chevalier C., Kalach N., Bergeret M., Labigne A., Dauga C. (2004). Genetic and transmission analysis of *Helicobacter pylori* strains within a family. Emerg. Infect. Dis..

[18] Falush D., Kraft C., Taylor N.S., Correa P., Fox J.G., Achtman M., Suerbaum S. (2001). Recombination and mutation during long-term gastric colonization by *Helicobacter pylori*: Estimates of clock rates, recombination size, and minimal age. Proc. Natl. Acad. Sci. USA.

[19] Morelli G., Didelot X., Kusecek B., Schwarz S., Bahlawane C., Falush D., Suerbaum S., Achtman M. (2010). Microevolution of *Helicobacter pylori* during prolonged infection of single hosts and within families. PLoS Genet..

[20] Cao Q., Didelot X., Wu Z., Li Z., He L., Li Y., Ni M., You Y., Lin X., Li Z. (2015). Progressive genomic convergence of two *Helicobacter pylori* strains during mixed infection of a patient with chronic gastritis. Gut.

[21] Linz B., Windsor H.M., McGraw J.J., Hansen L.M., Gajewski J.P., Tomsho L.P., Hake C.M., Solnick J.V., Schuster S.C., Marshall B.J. (2014). A mutation burst during the acute phase of *Helicobacter pylori* infection in humans and rhesus macaques. Nat. Commun..

[22] Lario S., Ramírez-Lázaro M.J., Sanjuan-Herráez D., Brunet-Vega A., Pericay C., Gombau L., Junquera F., Quintás G., Calvet X. (2017). Plasma sample based analysis of gastric cancer progression using targeted metabolomics. Sci. Rep..

[23] Schmieder R., Edwards R. (2011). Quality control and preprocessing of metagenomics datasets. Bioinformatics.

[24] Schloss P.D., Westcott S.L., Ryabin T., Hall J.R., Hartmann M., Hollister E.B., Lesniewski R.A., Oakley B.B., Parks D.H., Robinson C.J. (2009). Introducing mothur: Open-source, platform-independent, community-supported software for describing and comparing microbial communities. Appl. Environ. Microbiol..

