# Supplementary material for: Detection of Helicobacter pylori Microevolution and Multiple Infection from Gastric Biopsies by Housekeeping Gene Amplicon Sequencing
Source: Pathogens. 2020 Feb 5;9(2):97. doi: 10.3390/pathogens9020097 (PMC7168683; doi:10.3390/pathogens9020097)
Supplement: Supplementary file 1 [file pathogens-09-00097-s001.pdf]

**Supplementary Table S1.** Reference sequences used in this study.

| *H. pylori* strain | *cgt* sequence | *luxS* sequence | Observations |
|---|---|---|---|
| 7C | CP012905 | CP012905 | |
| 29CaP | CP012907 | CP012907 | |
| 35A | CP002096 | CP002096 | |
| 51 | CP000012 | CP000012 | |
| 52 | CP001680 | CP001680 | |
| 83 | CP002605 | CP002605 | |
| 908 | CP002184 | CP002184 | *cgt* and *luxS* sequences identical to these from the strain 2017 |
| 2017 | CP002571 | CP002571 | |
| 2018 | CP002572 | CP002572 | *cgt* and *luxS* sequences identical to these from the strain 2017 |
| 26695 | CP003904 | CP003904 | |
| 26695 (2) | NC_000915 | NC_000915 | *cgt* and *luxS* sequences identical to these from the strain 26695 |
| 26695-1 | AP013354 | AP013354 | *cgt* and *luxS* sequences identical to these from the strain 26695 |
| 26695-1MET | CP010436 | CP010436 | *cgt* and *luxS* sequences identical to these from the strain 26695 |
| Aklavik86 | CP003476 | CP003476 | |
| Aklavik117 | CP003483 | CP003483 | |
| APP134 | KU053359 | KU053431 | |
| ATCC 49503 | KU053360 | KU053432 | |
| ATCC 51932 | KU053361 | KU053433 | |
| B8 | NC_014256 | NC_014256 | |
| B38 | NC_012973 | NC_012973 | |
| B247 | KU053362 | KU053434 | |
| B271 | KU053363 | KU053435 | |
| B319 | KU053364 | KU053436 | |
| B355 | KU053365 | KU053437 | |
| B373 | MG950173 | MG950172 | |
| B491 | KU053366 | KU053438 | |
| B508S | KU053367 | KU053439 | |
| B657A-1 | KU053370 | KU053442 | |
| B657C | KU053372 | KU053444 | |
| B659A | KU053373 | KU053445 | |
| BM012A | CP006888 | CP006888 | |
| BM012B | CP007605 | CP007605 | *luxS* sequence identical to that from the strain BM012A |
| BM012S | CP006889 | CP006889 | *cgt* and *luxS* sequences identical to these from the strain BM012A |
| BM013A | CP007604 | CP007604 | |
| BM013B | CP007606 | CP007606 | *cgt* and *luxS* sequences identical to these from the strain BM013A |
| CC33C | CP011484 | CP011484 | |
| CRL122 | KU053375 | KU053447 | |
| Cuz20 | CP002076 | CP002076 | |
| ELS37 | CP002953 | CP002953 | |
| F16 | AP011940 | AP011940 | |
| F30 | AP011941 | AP011941 | |
| F32 | AP011943 | AP011943 | |

| | | | |
|---|---|---|---|
| F57 | AP011945 | AP011945 | |
| G27 | NC_011333 | NC_011333 | |
| Gambia94/24 | CP002332 | CP002332 | |
| Hp238 | CP010013 | CP010013 | |
| HPAG1 | NC_008086 | NC_008086 | |
| HUP-B14 | NC_017733 | NC_017733 | |
| India7 | CP002331 | CP002331 | |
| J99 | NC_000921 | NC_000921 | |
| J166 | CP007603 | CP007603 | *cgt* sequence identical to that from the strain B8 |
| L7 | CP011482 | CP011482 | |
| Lithuania75 | CP002334 | CP002334 | |
| ML1 | AP014710 | AP014710 | |
| ML2 | AP014711 | AP014711 | |
| ML3 | AP014712 | AP014712 | |
| OK113 | AP012600 | AP012600 | *cgt* sequence identical to that from the strain F32 |
| OK310 | AP012601 | AP012601 | |
| oki102 | CP006820 | CP006820 | |
| oki112 | CP006821 | CP006821 | *cgt* sequence identical to that from the strain oki102 |
| oki128 | CP006822 | CP006822 | |
| oki154 | CP006823 | CP006823 | |
| oki422 | CP006824 | CP006824 | *luxS* sequence identical to that from the strain oki112 |
| oki673 | CP006825 | CP006825 | *cgt* and *luxS* sequences identical to these from the strain oki128 |
| oki828 | CP006826 | CP006826 | *luxS* sequence identical to that from the strain oki128 |
| oki898 | CP006827 | CP006827 | *cgt* and *luxS* sequences identical to these from the strain oki112 |
| P12 | NC_011498 | NC_011498 | |
| PeCan4 | NC_014555 | NC_014555 | |
| PeCan18 | NC_017742 | NC_017742 | |
| Puno120 | CP002980 | CP002980 | |
| Puno135 | CP002982 | CP002982 | *cgt* sequence identical to that from the strain F32 |
| Rif1 | CP003905 | CP003905 | *cgt* and *luxS* sequences identical to these from the strain 26695 |
| Rif2 | CP003906 | CP003906 | *cgt* and *luxS* sequences identical to these from the strain 26695 |
| Sat464 | CP002071 | CP002071 | |
| Shi112 | NC_017741 | NC_017741 | *cgt* sequence identical to that from the strain Cuz20 |
| Shi169 | NC_017740 | NC_017740 | *cgt* sequence identical to that from the strain Sat464 |
| Shi417 | NC_017739 | NC_017739 | *cgt* sequence identical to that from the strain Sat464 |
| Shi470 | NC_010698 | NC_010698 | |
| SJM180 | NC_014560 | NC_014560 | |
| SNT49 | CP002983 | CP002983 | |
| SouthAfrica7 | CP002336 | CP002336 | |
| SouthAfrica20 | CP006691 | CP006691 | |
| SVC135 | KU053376 | KU053448 | |
| UM032 | CP005490 | CP005490 | *cgt* sequence identical to that from the strain ML2 |
| UM037 | CP005492 | CP005492 | |
| UM066 | CP005493 | CP005493 | *cgt* sequence identical to that from the strain ML3 |
| UM298 | CP006610 | CP006610 | *cgt* and *luxS* sequences identical to these from the strain UM032 |

| | | | |
|---|---|---|---|
| UM299 | CP005491 | CP005491 | *cgt* and *luxS* sequences identical to these from the strain UM032 |
| v225d | CP001582 | CP001582 | |
| XZ274 | CP003419 | CP003419 | |
